# Novel metagenomics analysis suggests a *Vibrio* species is associated with stony coral tissue loss disease

**DOI:** 10.1101/2024.01.02.573916

**Published:** 2024-01-03

**Authors:** Jakob M. Heinz, Markus Sommer, Stephanie M. Rosales, Jennifer Lu, Lindsay K. Huebner, Steven L. Salzberg

**Affiliations:** 1 Center for Computational Biology, Johns Hopkins University; Baltimore, MD 21218, United States; 2 Department of Biomedical Engineering, Johns Hopkins School of Medicine and Whiting School of Engineering; Baltimore, MD 21218, United States; 3 Cooperative Institute for Marine and Atmospheric Studies, University of Miami; Miami, FL 33149, United States; 4 Atlantic Oceanographic and Meteorological Laboratory, National Oceanographic and Atmospheric Administration, Miami, FL 33149, United States; 5 Department of Pathology, Johns Hopkins School of Medicine, Baltimore, MD 21218, United States; 6 Fish and Wildlife Research Institute, Florida Fish and Wildlife Conservation Commission; St. Petersburg, FL 33701, United States; 7 Department of Computer Science, Johns Hopkins University; Baltimore, MD 21211, United States; 8 Department of Biostatistics, Johns Hopkins University; Baltimore, MD 21211, United States

**Keywords:** Bioinformatics, Metagenomics, SCTLD, Coral reef, Epidemic

## Abstract

Stony coral tissue loss disease (SCTLD) has devastated coral reefs off the coast of Florida and continues to spread throughout the Caribbean. Although a number of bacterial taxa have consistently been associated with SCTLD, no pathogen has been definitively implicated in the etiology of SCTLD. Previous studies have predominantly focused on the prokaryotic community through 16S rRNA sequencing of healthy and infected tissues. Here, we provide a different analytical approach by applying a bioinformatics pipeline to publicly available whole genome sequencing samples of SCTLD lesions and healthy tissues from four stony coral species. To compensate for the lack of coral reference genomes, we used data from apparently healthy coral samples to approximate a host genome and healthy microbiome reference. The healthy reference reads were then used to filter the reads from diseased lesion tissue samples, and the remaining data were taxonomically classified at the DNA and protein levels. For DNA classifications, we used a pathogen identification protocol originally designed to identify pathogens in human tissue samples, and for protein classifications, we used a fast protein sequence aligner. Although these data were previously analyzed, our approach revealed unique patterns that were not identified in the previous work. We found a relatively high abundance of the *Vibrio* genus across diseased samples as well as a number of enriched *Vibrio* phages that further support the presence of this genus in diseased samples, suggesting that a member of the *Vibrio* genus may be involved in the visual lesion formation stage of SCTLD.

## Article Summary

Studies of stony coral tissue loss disease (SCTLD), a devastating coral disease, have primary used 16S rRNA sequencing approaches to identify putative pathogens. This study applied human tissue pathogen identification protocols to SCTLD whole genome DNA samples. A k-mer based filtering method for diseased samples was utilized to compensate for the lack of host reference genomes. DNA and protein level classifications revealed a relatively abundant member of the *Vibrio* genus associated with SCTLD across four stony coral species.

## Introduction

Stony coral tissue loss disease (SCTLD) was discovered off the coast of Miami, FL in 2014 and has since had negative consequences on the function of coral reefs across Florida and the Caribbean (Walton *et al*. 2018; Alvarez-Filip *et al*. 2022). To date, despite many efforts, no pathogen has been definitively identified as the causative agent of SCTLD. The stony coral (order Scleractinia) microbiome is a complex system of interactions between the host, bacteria, viruses, fungi, archaea, and algal symbionts (Bourne *et al*. 2009); thus a disturbance in any number of these symbiotic relationships could be involved in SCTLD progression. Multiple studies have explored viruses that may infect stony coral symbionts, notably *Symbiodiniaceae*, but no causative relationships have been detected (Work *et al*. 2021; Veglia *et al*. 2022; Beavers *et al*. 2023; Howe-Kerr *et al*. 2023). Bacterial species are particularly under scrutiny for their potential involvement in SCTLD, due to the effectiveness of antibiotics in halting lesion progression in multiple affected coral species (Neely *et al*. 2020; Shilling *et al*. 2021; Studivan *et al*. 2023). Consequently, SCTLD studies have predominantly focused on understanding changes in the bacterial community between apparently healthy and SCTLD-affected corals.

Studies to identify bacterial pathogens have relied primarily on small subunit 16S ribosomal RNA (rRNA) sequencing, followed by computational analysis (Callahan *et al*. 2016) typically using the Silva database (Quast *et al*. 2013) to assign and classify Amplicon Sequence Variants (ASVs) into taxa. ASVs found in diseased lesion samples are then compared to samples from apparently healthy colonies to determine which ASVs are associated with the tissue loss lesions (Meyer *et al*. 2019; Rosales *et al*. 2020; Clark *et al*. 2021). These methods have characterized many notable shifts in coral bacterial communities due to SCTLD and identified a number of bacterial taxa have been associated with SCTLD, including *Rhizobiales*, *Clostridiales*, *Peptostreptococcales-Tissierellales*, *Rhodobacteraceae*, *Flavobacteriaceae*, and *Vibrionaceae* (Rosales *et al*. 2023). However, because of the difficulty in determining whether an associated bacterial taxon is a harmless commensal, an opportunistic secondary infection, or the primary pathogen, none of the bacterial taxa associated with SCTLD have been identified as the causative agent.

An alternative approach to understanding disease dynamics is the use of metagenomic whole-genome sequencing (WGS), in which all of the DNA from a source is sequenced, including not only the host, but also viruses, bacteria, and eukaryotic species living on or within the host tissue. For example, by analyzing WGS data of human tissue samples taken from the site of infection, researchers have identified pathogenic agents in brain infections (Salzberg *et al*. 2016; Wilson *et al*. 2019), corneal infections (Eberhart *et al*. 2017), and other diseases (Kostic *et al*. 2012). The sensitivity of this approach relies on first, sequencing the source DNA deeply enough to capture the pathogen of interest, and second, the existence of closely related host genomes with sequence similarity to the pathogen in the public databases. While the number of complete genomes has grown enormously over the past two decades, databases still contain few or no genomes for non-model organisms, including scleractinian corals.

Currently, only one SCTLD metagenome study with WGS data is publicly available. While the authors of that study (Rosales *et al*. 2022) were able to assemble and annotate genomes for SCTLD-associated bacterial taxa such as *Rhodobacterales*, *Rhizobiales*, and *Flavobacteriales*, the results were focused on only five of the twenty diseased lesion tissue samples, and the majority of samples were dominated by host sequences. In metagenomic studies, host sequences can confound results, so they are typically removed by aligning the reads to a host reference genome. Currently, the GenBank database has 53 genome assemblies from scleractinian corals, of which only seven are at the chromosome level (NCBI 2023). Of these 53 genomes, none are from the species of corals previously investigated for SCTLD (Rosales *et al*. 2023), emphasizing the additional challenges associated with using metagenomics in non-model organisms. Additionally, given the complex symbiotic microbiome (i.e., algal symbiont, viruses, and prokaryotic community) of stony corals (Bourne *et al*. 2009), the host DNA is only one of the hurdles.

In this study, we applied new classification methods to understand this devastating coral disease. We used a method to filter host reads from metagenome data by using data collected from apparently healthy corals of the same species to approximate a species-specific healthy host coral genome and microbiome. We then applied the Kraken software suite for pathogen identification (Lu *et al*. 2022) using KrakenUniq (Breitwieser *et al*. 2018) with the goal of identifying putative pathogens present in diseased samples and not present in healthy ones. Using these methods, we identified a number of taxa that have previously been associated with SCTLD, providing further support for their involvement in SCTLD pathogenesis. In addition, in diseased samples from all four coral species investigated here, we found an elevated abundance of species from the *Vibrio* genus, suggesting the *Vibrio* genus is associated with the visual lesion formation stage in SCTLD.

## Methods

### Data acquisition

We downloaded 58 WGS datasets from NCBI Bioproject PRJNA576217 (Benson *et al*. 2017), previously generated by Rosales *et al*. (Rosales *et al*. 2020, 2022). Sample SRR15960000, an apparently healthy *Diploria labyrinthiformis* sample, was removed due to data quality problems, leaving 57 sets of paired-end samples for analysis. These were 20 diseased colony lesion (DL) samples, 20 diseased colony unaffected (DU) samples, and 17 apparently healthy colony (AH) samples from the coral species *D*. *labyrinthiformis*, *Dichocoenia stokesii*, *Meandrina meandrites*, and *Stephanocoenia intersepta*. DU samples were taken from apparently unaffected tissue from the diseased corals also sampled for DL. All samples were from corals within reefs with an ongoing SCTLD outbreak in the Florida Keys. Consequently, it is possible that a primary pathogen of SCTLD could be present in low abundance in at least one of the AH samples or that the AH microbiome was different from that of corals in reefs where SCTLD had yet to arrive. Therefore, in this study, our findings represent microbial communities enriched in the observable surface tissue loss formation stage of SCTLD (hereafter visual tissue loss) compared to corals with no visual signs of disease (i.e., AH).

The tissue samples from the four coral species were pooled by each of the three disease states, resulting in twelve pooled read files (AH, DU, and DL for each of the four coral species). It was assumed that a putative pathogen involved in visual tissue loss would likely show different abundances in DL samples during different stages of lesion progression, so pooling the samples was thought to increase the likelihood of observing a putative agent. All subsequent analyses were based on these data, focusing primarily on the DL and AH samples. The reads from the DU samples were explored in the preliminary analysis, but were not considered in the final analysis. Due to the proximity of the DU samples to lesion tissue, DU samples were considered likely to represent early stages of surface tissue loss, and therefore poor choices for our methods.

### Filtering reads with a healthy coral reference database

**Figure 1:**
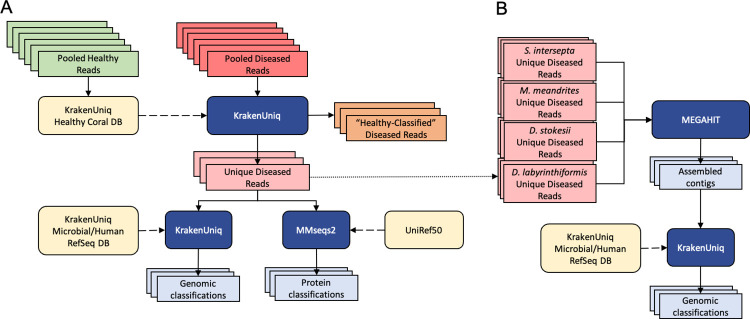
**(A)** Filtering the diseased reads consisted of building a KrakenUniq database from all healthy reads for every coral species and classifying the corresponding pooled diseased reads against this database. Reads that were unclassified by the database were considered unique to the diseased samples. This subset was classified at the DNA level with KrakenUniq and at the protein level with MMseqs2. **(B)** The unique diseased reads from all coral species were combined and assembled with MEGAHIT. The assembled contigs were then classified with KrakenUniq.

Because no sequenced genome was available for any of the four coral species, we created a customized database to identify reads that likely originated from either the host genome or the healthy host microbiome. To do this, we used reads from all AH samples to create a KrakenUniq (Breitwieser *et al*. 2018) database for each coral species. We then used this database along with KrakenUniq to classify reads from DL samples, thereby removing any reads in DL samples that matched any read in the AH samples. This filtering step produced a subset of diseased reads that we considered unique to the DL samples, and greatly reduced the number of reads analyzed in subsequent steps (Figure 1A).

The k-mer size for databases was set to 29bp, lower than the default of 31bp because we wanted to filter more aggressively. For all other parameters, the default values of KrakenUniq were used. For each coral species, the pooled DL reads were then classified with KrakenUniq against the AH reads database corresponding to that species. The original DL files were parsed to extract all reads that were unclassified by KrakenUniq, providing us with the files used in the subsequent analysis (Figure 1A). With this aggressive filtration approach, we may have lost information about changes in relative abundances between the AH and DL samples, but we were left with reads that were truly unique to the DL samples. This set would likely provide the clearest signal of microbes associated with visual lesion formation, which was the primary goal of this study.

### KrakenUniq read classification

The unique DL reads were first classified with KrakenUniq (Breitwieser *et al*. 2018) using default parameters and the default k-mer size of 31 bps against a microbial database (Figure 1A). The database used for classification was built in August 2020 using all NCBI RefSeq complete bacterial, viral, and archaeal genomes, the GRCh38 human genome, the NCBI UniVec database, and a curated set of sequences from EuPathDB (Lu and Salzberg 2018; Amos *et al*. 2022). If there are novel species associated with SCTLD, then their genomes will not be present in public databases; however, if closely related species from the same genera are available, then we might find DNA sequence-level matches to those genomes. For this reason, the unique k-mers are reported at the genus level. Additionally, the relative abundances of the genera are calculated by the unique k-mer count rather than the read count. In general, using the unique k-mer count (i.e., sequences of length k are counted just once per taxon, no matter how many times they occur in the raw data) rather than read count reduces the bias introduced from using amplification-based sequencing workflows. Using the unique k-mer count also reduces false positives that may arise from reads that contain low-complexity k-mers (Breitwieser *et al*. 2018).

The report files were initially visualized and explored with Pavian (Breitwieser and Salzberg 2020). The read classifications were verified by randomly sampling classified reads, aligning them with megablast (Altschul *et al*. 1990) to standard databases, and ensuring they had the same or similar classifications as with KrakenUniq. The unique k-mers-per-read statistic served as a confidence flag. For a species that was truly present in the sample, even with amplified WGS data, we expected a high number of unique k-mers per read. A 150 bp read may contain up to 120 unique 31-mers, although repetitive k-mers will reduce the unique count. There is also an upper bound to unique k-mers found in a genome, which may be reached when the genome is small or when the sampling depth is high. In this study, we considered a value of less than five unique k-mers per read as a flag that the taxon might be a false positive. The unique k-mer-per-read count for every classified genus is reported in Supp. Table S1.

### MMseqs2 read classification

Because protein sequences are more conserved than DNA across distant species, we ran translated searches using MMSeqs2 easy-taxonomy workflow (Steinegger and Söding 2017) with the UniRef50 protein database (Suzek *et al*. 2015) to determine if this would identify more of the microbial reads than DNA sequences alone (Figure 1A). The protein database used was UniRef50 (Suzek *et al*. 2015), which allows for faster alignment given that we aligned our protein sequences to clusters of similar protein sequences rather than all protein sequences. The paired reads had to be classified separately because MMseqs2 easy-taxonomy does not allow for both paired reads to be processed together.

To identify reads belonging to members of the algal symbiont family *Symbiodiniaceae*, the MMseqs2 output was parsed to extract all protein cluster identifiers that had at least one alignment at the “f_Symbiodiniaceae” level or below for each coral species. The UniRef50 cluster identifiers were mapped to the full UniProtKB (UniProt Consortium, 2021). Their functions, if known, are reported in Supp. Table S2 as output by the UniProt ID mapping service (Huang *et al*. 2011). Supp. Table S2 was produced by inserting the number of alignments from the original MMseqs2 “tophit_report” files into the outputs of the UniProtKB ID mapping.

### MEGAHIT contig assembly and classification

Due to the high genomic diversity of viruses (Aiewsakun *et al*. 2018), a viral agent might have been missed in our previous analyses because it was too divergent from available DNA and protein sequences. This problem could be mitigated if the query sequences were longer, and therefore we assembled the raw reads to see if any long viral contigs were assembled.

The filtered unique diseased reads from all four coral species were pooled to form a fasta file of all filtered unique diseased reads and were then assembled with MEGAHIT v1.2.9 (Li *et al*. 2016) using default parameters (Figure 1B). The contigs were classified with KrakenUniq using the same database of complete bacterial and viral genomes used above. The viral classifications from the report file were extracted to search for any viruses of interest. These steps were repeated with pooling just the filtered unique diseased reads excluding *S*. *intersepta* because samples from this species represented a majority of the reads (89.8%) in our study and dominated the previous assembly (Rosales *et al*. 2022).

To investigate virulence factors associated with *Vibrio*, a genus implicated in other coral diseases as well as SCTLD (Munn 2015; Meyer *et al*. 2019), and found in high abundance in this study (see Results), the 9,427 contigs classified as *Vibrio* from the MEGAHIT assembly with all four coral species were extracted. These contigs were then aligned with megablast (Altschul *et al*. 1990) to the Virulence Factor Database (VFDB) DNA sequences core dataset (Liu *et al*. 2022), downloaded on May 12th, 2023.

### Comparison to cultured *Vibrio* assemblies from a prior SCTLD study

The reads that were classified at or below the *Vibrio* genus level by KrakenUniq were extracted from the original sequence files for *S*. *intersepta* and *D*. *labyrinthiformis* only, because they contributed the majority of the *Vibrio* genus reads. The draft genomes from an SCTLD study that cultured *V*. *coralliilyticus* (Ushijima *et al*. 2020) were downloaded from NCBI Bioproject PRJNA625269 (Benson *et al*. 2017) and a Bowtie2 index was built for each one. The extracted *Vibrio* reads were aligned with Bowtie2 (Langmead and Salzberg 2012) to each of the eight draft genomes. The Bowtie2 alignment rates to each draft genome are reported in Supp. Table S4.

### *Vibrio* species analysis

Given the interest in the *Vibrio* genus, the reads that were classified at the species level were investigated in further detail. The KrakenUniq report files of the unique diseased reads for every coral species were parsed to extract the number of unique k-mers assigned to each *Vibrio* species. The k-mer counts were normalized by dividing them by the total number of k-mers assigned to the genus. The contribution of *Vibrio* species reads from each sample was then found by parsing the KrakenUniq output to determine the sample ID number from the read identifier. It was not possible to determine the number of unique k-mers that were contributed by each sample with these methods.

## Results

We initially analyzed whole-genome sequencing reads from *D*. *labyrinthiformis*, *D*. *stokesii*, *M*. *meandrites*, and *S*. *intersepta* from three different tissue sample types: diseased colony lesion (DL), diseased colony unaffected (DU), and apparently healthy colony (AH). The total number of reads from each species and sample type is shown in Table 1.

### Genus level classification with KrakenUniq

**Figure 2:**
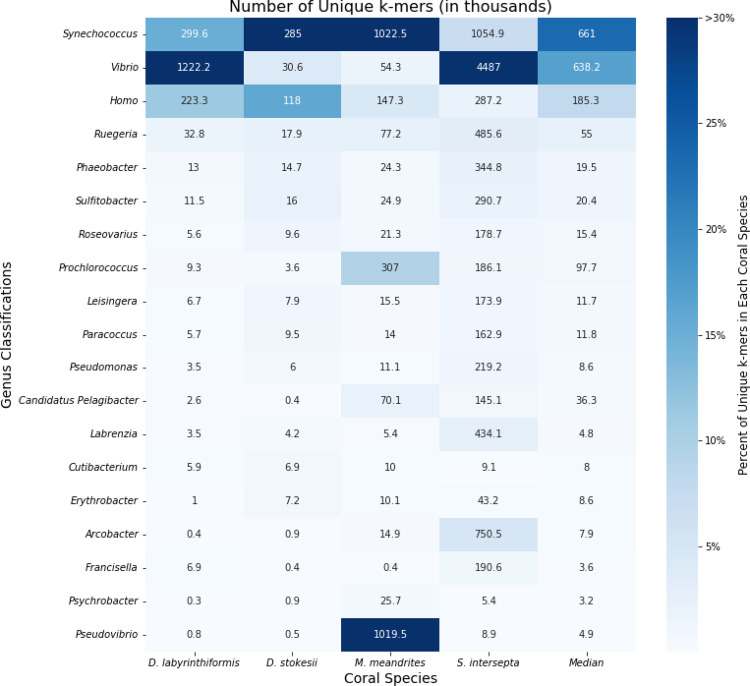
Unique k-mer counts from KrakenUniq genus-level classifications of unique diseased reads for every coral species. The intensity of the shading represents the percent of total unique k-mers assigned to the genus. Each box is annotated with the number of unique k-mers (in thousands) assigned to the genus.

When the KrakenUniq reports were sorted by unique k-mer count within coral species, the six genera *Synechococcus*, *Vibrio*, *Homo*, *Ruegeria*, *Phaeobacter*, and *Sulfitobacter* were among the ten most differentially abundant in all coral species, with the genera *Vibrio*, *Synechococcus*, and *Ruegeria* being particularly abundant across all coral species (Figure 2). *Synechococcus* was the most or second most abundant in all coral species. In *D*. *labyrinthiformis*, *Vibrio* was the most abundant, with 4.1 times more unique k-mers than the second most abundant genus, *Synechococcus*. In *D*. *stokesii*, *Synechococcus* was the most abundant with *Homo* and *Vibrio* being second and third most abundant, respectively (note that *Homo* is due to human contaminants). In *M*. *meandrites*, the *Synechococcus* genus was again the most abundant. The *Pseudovibrio* genus was the second most abundant in *M*. *meandrites*, but only appeared as substantially differentially abundant in this coral species. *Vibrio* had the seventh highest relative abundance in *M*. *meandrites*, which, though lower than observed in the other coral species, still represented a high k-mer-to-read ratio of 52.0 (Supp. Table S1). In *S*. *intersepta*, the *Vibrio* genus was again clearly the most abundant, having 4.3 times the amount of unique k-mers compared to the second place *Synechococcus*. Due to their high abundances across all coral species in this analysis, *Vibrio*, *Synechococcus*, and the *Rhodobacteraceae* family (to which *Ruegeria*, *Phaeobacter*, and *Sulfitobacter* belong) appear to be associated with visual tissue loss and may represent important agents of pathogenesis in SCTLD.

### Protein-level classification with MMseqs2

The number of microbial reads classified for each coral species at the protein-level using MMseqs2 (Steinegger and Söding 2017) increased approximately six-fold compared to the DNA-based searches (Supp. Figure S1). Because we were primarily interested in whether any new candidate taxa emerged, we did not consider the relative abundances of different taxa classified by the protein-based search compared to the DNA-based search.

**Figure 3.**
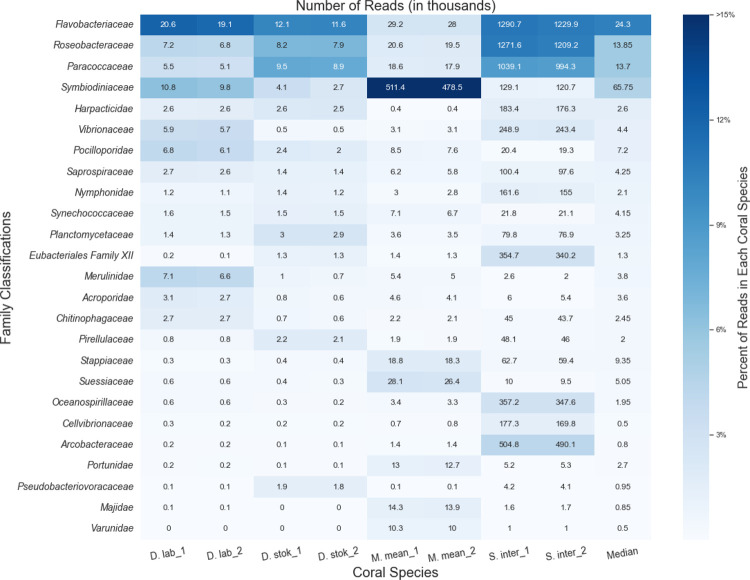
Classifications from MMseqs2 at the family-level for forward (“1”) and reverse reads (“2”) from unique diseased reads from each coral species. The intensity of the shading represents the proportion of total reads from the coral species that were assigned to the family. The boxes are annotated with the number of reads (in thousands) assigned to each family.

These results are shown in Figure 3. Note that MMSeqs2 does not handle paired reads as a unit, so the paired-end reads were classified separately. As expected, the read counts were similar between the paired reads for every coral species. Additionally, MMSeq2 does not report k-mer counts, only read counts, a metric which is subject to more bias from PCR amplification protocols, as explained above. Due to the decreased specificity of a protein search, MMseqs2 classified many reads as “unclassified [family level]”; thus, the results are presented at the family level rather than the genus level in Figure 3.

MMseqs2 was able to classify a higher percentage of bacterial and eukaryotic reads, predominantly from coral algal symbionts such as *Symbiodiniaceae*, yet we saw similar abundant taxa as in the KrakenUniq analysis. *Symbiodiniaceae* was among the top families in all coral species and particularly abundant (~48% of all classified reads) in *M*. *meandrites*. Due to particular interest in the role of *Symbiodiniaceae* in SCTLD progression (Beavers *et al*. 2023), the functions of the proteins in the *Symbiodiniaceae* protein clusters identified are provided in Supp. Table S2.

The families *Flavobacteriaceae*, *Roseobacteraceae*, and *Paracoccaceae* were among the most abundant families in all coral species. In this database, *Roseobacteraceae* and *Paracoccaceae* are homotypic synonyms of the family *Rhodobacteraceae* in the database used for DNA-level classifications (Göker 2022). So, we observed *Rhodobacteraceae* as before in the DNA analysis, but *Flavobacteriaceae* emerged as another family of interest in this protein-level analysis.

*Flavobacteriaceae* was also found as a top family in the DNA-level classification, however, no top genus was identified that belongs to this family. Additionally, the average number of unique k-mers stemming from reads classified at or below the *Flavobacteriaceae* family at the DNA-level was relatively low. For example, in *S*. *intersepta*, there were 794,258 unique k-mers from 489,794 reads, or ~1.6 k-mers per read. In *D*. *stokesii* there were 38,333 unique k-mers from 3,802 reads, or ~10 k-mers per read. Across all coral species, the *Flavobacteriaceae* family had one of the lowest average k-mer-per-read counts of all bacterial families identified. For example, the *Vibrionaceae* family, which has similar sized genomes to *Flavobacteriaceae* (Lin et al. 2018; Gavriilidou et al. 2020), had 25 k-mers-per-read and 88 k-mers-per-read in *S*. *intersepta* and *D*. *stokesii*, respectively.

### Contig Assembly and Classification

**Figure 4:**
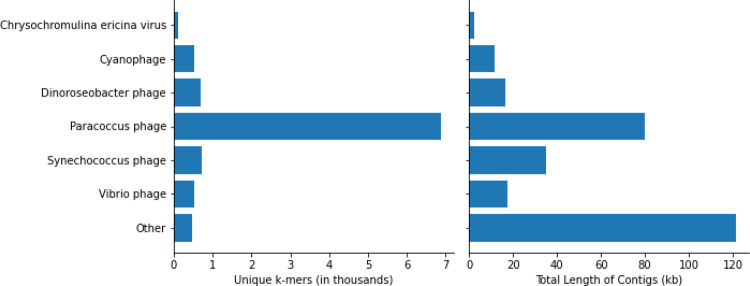
Classifications of assembled viral contigs showing the number of unique k-mers (in thousands) and the total sequence length (in kb) assembled for every genus.

In addition to characterizing the bacterial community, using metagenomic WGS data made it possible to explore DNA viruses found in the unique DL reads. To account for the genomic diversity of viruses, which may not share many conserved sequences with genomes in public databases, we assembled contigs from the unique DL reads and classified them with KrakenUniq to identify any viral contigs that may be of interest in SCTLD etiology. This resulted in 1,014,402 assembled contigs. KrakenUniq classified 168,829 (16.6%) contigs, of which only 227 (0.02%) were viruses. The viral contig classifications are shown in Figure 4. *Paracoccus* phages were the most abundant, with *Vibrio* phages, *Synechococcus* phages, *Dinoroseobacter* phages, and *Cyanophages* being abundant as well. Five contigs with 108 unique k-mers were classified as *Chrysochromulina ericina* virus, a virus that infects the microalga *Chrysochromulina ericina* (also known as *Haptolina ericina*) (Gallot-Lavallée *et al*. 2017).

When combining the filtered diseased reads from every coral species for assembly, the reads originating from *S*. *intersepta* samples represented a majority of the reads (89.8%). Therefore, we repeated the previous steps without *S*. *intersepta* reads and assembled the reads from the other three coral species. This resulted in 27,759 assembled contigs, of which 2,010 (7.2%) were classified by KrakenUniq, and only six matched viruses (five *Pseudoalteromonas* phages and one *Synechococcus* phage).

The contig assembly results indicate that there is likely not an abundant DNA virus of interest in the diseased samples. A virus that infects the microalga *Chrysochromulina ericina* was detected, however with few unique k-mers (108) and with only 2.5 kb assembled of a 473.6 kb genome (Gallot-Lavallée *et al*. 2015). The five *C*. *ericina* virus contigs were aligned with BLASTN (Altschul *et al*. 1990) to standard databases, which revealed that while two contigs aligned best to *C*. *ericina* virus, the other three aligned best to Eukarya, possibly indicating false positives and leading us to be skeptical of any significant implications of this finding for SCTLD progression. Primarily, this analysis detected phages of bacteria that were found in high abundances in the results above. The detection of these phages provided support that the high abundances of the associated bacteria previously observed were representative of the true metagenomic compositions of the samples.

### *Vibrio* Species Classifications

**Figure 5.**
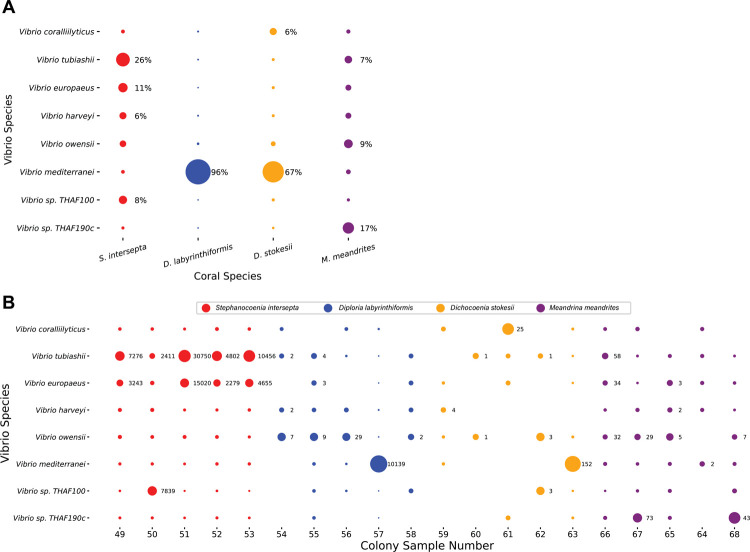
Species-level KrakenUniq classifications of *Vibrio* genus reads in SCTLD lesions. (**A**) The proportion of unique k-mers of all *Vibrio* species pooled from each coral species. The size of the dots is relative to the proportion of unique k-mers assigned to the *Vibrio* species in each coral species. Those with over 5% are annotated with their proportion. (**B**) The species level proportions of the *Vibrio* genus reads for every sample. The x-axis is labeled with the colony sample number, which corresponds to those assigned in the original analysis of this dataset (Rosales *et al*. 2020). *Vibrio* species classifications that represent at least 5% of the *Vibrio* species in the sample are annotated with the number of reads.

The results presented above identified the *Vibrio* genus as a strong candidate agent of surface tissue loss. Given that a number of V*ibrio* species have been associated with other coral pathogens (Munn 2015), we were particularly interested in this genus. We also found that various assembled *Vibrio* contigs contained virulence factors (locations and functions in Supp. Table S3). This indicated that the *Vibrio* identified had the genetic potential to be pathogenic in the visual tissue loss stage. Therefore, we investigated the *Vibrio* classifications at the species level. The proportion of all *Vibrio* genus unique k-mers contributed by each *Vibrio* species are displayed in Figure 5A and the proportion of all *Vibrio* genus reads contributed by each sample is shown in Figure 5B.

*V*. *europaeus* and *V*. *tubiashii*, two closely related species, represented a large portion (37% combined) of the *Vibrio* k-mers in *S*. *intersepta* (Figure 5A). *V*. *mediterranei* dominated in *D*. *labyrinthiformis* (96%) and *D*. *stokesii* (67%) but was muted in the other coral species (Figure 5A); however, one colony per species appeared to be responsible for these high proportions: colony 57 in *D*. *labyrinthiformis* and colony 63 in *D*. *stokesii* (Figure 5B). *V*. *sp*. *THAF190c* was the predominant species (17%) in *M*. *meandrites*. Other species like *V*. *coralliilyticus*, *V*. *harveyi*, *V*. *owensii*, and *V*. *sp*. *THAF100* appeared consistently in all coral species, but never in high proportions. As seen in Figure 5B, *V*. *tubiashii* and *V*. *owensii* contributed more than 5% of the reads in 10 samples each.

### Comparison to cultured *Vibrio* assemblies from a prior SCTLD study

Eight draft genomes from *V*. *coralliilyticus* strains isolated from a previous SCTLD study (Ushijima *et al*. 2020) allowed us to compare whether we identified the same *Vibrio* species. *Vibrio* genus reads classified by KrakenUniq in *S*. *intersepta* and *D*. *labyrinthiformis* were extracted and aligned to the Ushijima *et al*. draft genomes. Between 3.76% to 4.02% of the reads mapped to the *V*. *coralliilyticus* strains, while 7.54% of reads mapped to the McD22-P3 strain, which was the control strain, and not *V*. *coralliilyticus* (Supp. Table S4). As would be expected, the proportions of reads that aligned are similar to the proportions of reads that were classified as *V*. *coralliilyticus* by KrakenUniq (See Figure 5).

## Discussion

In this study we used previously published sequencing data from coral affected by SCTLD and developed a novel metagenomic analysis pipeline to explore the microbial communities present in those data. The data consisted of samples from four coral species collected from Florida’s coral reefs during a SCTLD outbreak. To investigate the microbial taxonomy of these samples, the previous study used small subunit rRNA gene assemblies and metagenome-assembled genomes. Our investigation differed by focusing on whole-genome shotgun data which we used along with the Kraken software suite for potential pathogen identification. Our analysis provides new insights that point to *Vibrio* as being associated with SCTLD. Previous work also did not filter out host sequences, but here we applied a novel technique to filter host reads from metagenomic data by using data derived from apparently healthy samples as a surrogate for a reference genome. This allowed us to examine unique sequences from DL samples by approximating a species-specific host coral genome. In addition, in this study, we investigated the SCTLD DNA virome, which has not been previously reported.

In our protein analysis, the families *Rhodobacteraceae* and *Flavobacteriaceae* were found to be associated with SCTLD, consistent with previous SCTLD studies. *Rhodobacteraceae* is one of the most common bacterial families associated with coral diseases (Gignoux-Wolfsohn *et al*. 2017) in diverse geographic locations, but no member has been identified as a causative coral disease agent (Mouchka *et al*. 2010), likely indicating their ability to opportunistically infect diseased coral. In addition to SCTLD (Rosales *et al*. 2023), *Flavobacteriaceae* has been enriched in White Band Disease in the scleractinian staghorn coral *Acropora cervicornis* (Gignoux-Wolfsohn and Vollmer 2015), but has never been identified as a causative agent in coral tissue loss. Additionally, our methods did not detect a single highly abundant genus belonging to *Flavobacteriaceae* in the unique diseased reads. For these reasons, members of *Flavobacteriaceae* within SCTLD DL tissue also most likely represent opportunistic infections. Although *Vibrionaceae* and *Synechococcaceae* were not among the most abundant families within the unique DL reads in the MMSeqs2 protein analysis (also the case for *Vibrionaceae* in the DNA-level classifications at the family level read counts), we were primarily interested in whether new candidates emerged from the MMSeqs2 analysis, not the relative abundance of the candidates. Therefore, the two genera within these two families that were abundant in the DNA-level analysis remained strong candidates for putative pathogens, which are assumed to have a high abundance at the genus level. In contrast, a family that appeared as highly abundant in the unique DL reads, without a single highly abundant genus, as we observed with *Flavobacteriaceae* and *Rhodobacteraceae*, may be more likely to represent a microbial group with multiple members capable of opportunistic infection of already-diseased coral tissue.

In our k-mer analysis, the genera *Synechococcus* and *Vibrio* were identified as taxa of interest, but interestingly were not detected in the previous analysis of this data. *Synechococcus*, the most or second most abundant genus across all four coral species, belongs to the phylum *Cyanobacteria*, which are photosynthetic picoplankton (Kim *et al*. 2018). Although not likely involved in pathogenesis, *Synechococcus* have been enriched in other SCTLD studies, and it has been hypothesized that their increase in abundance is a response to disease stress (Rosales *et al*. 2023). The high differential abundance of *Synechococcus* in this study supports the suggestion that *Synechococcus* may have some role in microbial community interactions during SCTLD. In contrast to *Synechococcus*, *Vibrio* have been associated with other coral tissue loss diseases, and have been found to cause coral bleaching (Munn 2015) unlike the other abundant bacterial taxa in this study. In three coral species, *Vibrio* was either the most abundant (two species) or third most abundant (one species) genus, making it a strong candidate for an agent involved in SCTLD visual tissue loss; In the fourth species, *M*. *meandrites*, *Vibrio* was only the seventh most abundant genus, however, this coral species had the fewest AH reads to create the database used for filtering the DL reads (Table 1), which may have led to more noise in the *M*. *meandrites* results compared to other coral species. Species belonging to the *Vibrio* genus are not always pathogenic, but we found various virulence factors, such as motility, adherence, and effector delivery systems, that further suggest the *Vibrio* from this study have the capacity to be pathogenic.

Within the *Vibrio* genus, many reads were classified to the species level, with matches to *V*. *coralliilyticus*, *V*. *harveyi*, and *V*. *owensii*, which are known coral pathogens associated with bleaching and tissue loss (Munn 2015). *V*. *mediterranei*, which comprised a majority of the classified *Vibrio* species in *D*. *labyrinthiformis* and *D*. *stokesii*, is 97% identical to *V*. *shilonii*, resulting in them now being considered the same species (Tarazona *et al*. 2014). *V*. *mediterranei*/*shilonii* were found to be responsible for the annual bleaching of the scleractinian coral *Oculina patagonica* off the Israeli coast from 1993–2003. Additionally, a more recent analysis of *O*. *patagonica* bleaching in the Spanish Mediterranean found that while *Vibrio* species were always present in healthy corals, *V*. *coralliilyticus* and *V*. *mediterranei*/*shilonii* were only identified in diseased corals. Although rarely identified together in wild samples, experiments found that introducing *V*. *coralliilyticus* and *V*. *mediterranei*/*shilonii* together to healthy corals was substantially more detrimental to their health than either of the species introduced alone (Rubio-Portillo *et al*. 2014). V. *mediterranei*/*shilonii*, therefore, may be of particular interest in future SCTLD studies, given its association with bleaching events and its high abundance in two coral species investigated in our study, paired with co-occurrence with *V*. *coralliilyticus* (although in differing proportions; Figure 5).

Our results are not the first to identify *Vibrio* associated with SCTLD (Ushijima *et al*. 2020; Rosales *et al*. 2023). Previous work has shown that *Vibrio* are enriched in SCTLD samples, but these studies did not find them as prevalent across samples, including in this dataset (Rosales *et al*. 2020). A study that cultured *V*. *coralliilyticus* from SCTLD lesions concluded that isolates did not cause disease, but if the *V*. *coralliilyticus* zinc-metalloprotease was detected, it was correlated with higher rates of both mortality and disease progression (Ushijima *et al*. 2020). The lack of similarity between those cultured *V*. *coralliilyticus* sequences and our *Vibrio* sequences leads us to believe that multiple *Vibrio* species may be involved in SCTLD lesion development. However, it is important to note that our picture of the SCTLD microbiome is restricted by the genomes in the databases used. The *Vibrio* genus has been found to have a large degree of genomic flexibility (Heidelberg *et al*. 2000), so while the classifications to different *Vibrio* species may truly represent the presence of an array of *Vibrio* species, it may instead be the result of various reads from a novel *Vibrio* species matching different *Vibrio* species based on closest genomic similarity. Therefore, while matches to different *Vibrio* species and their potential role in SCTLD may offer some insights, a more robust interpretation is to consider the implications of disease association by *Vibrio* at the genus level.

Some work has suggested that the etiology of SCTLD has been hypothesized to be viral (Robertson *et al*. 2023), and gene expression data show that there is an increase in coral viral immune response in corals with SCTLD (Beavers *et al*. 2023). Researchers have explored the potential role of RNA viruses in SCTLD, but no RNA viruses have been found enriched in corals with SCTLD (Veglia *et al*. 2022) and these viruses are likely ubiquitous in corals without any potential relationship to SCTLD (Howe-Kerr *et al*. 2023). To further investigate the involvement of viruses in SCTLD, we analyzed the presence of DNA viruses. Our data show the majority of DNA viruses in diseased samples represent phages. Not surprisingly, phage sequences correspond with some of the most abundant bacteria identified in this study, such as *Rhodobacteraceae*, *Vibrionaceae*, and *Synechococcaceae*. The *Paracoccus* phage, which infects *Rhodobacteraceae*, and the *Vibrio* phage would be interesting to further explore as potential avenues for disease mitigation. In addition to phages, sequences were found with similarities to the *Chrysochromulina ericina* virus. However, with only two contigs and little coverage of its genome, we do not believe this virus plays a role in SCTLD. Thus, we did not find any DNA viruses with definitive association with SCTLD. Future studies may consider viral enrichment protocols prior to sequencing to help better characterize the SCTLD DNA virome.

In addition to differences in the bacterial and viral communities, members of *Symbiodiniaceae* were differentially abundant in diseased samples compared to healthy samples. SCTLD disrupts the relationship between the host coral and its *Symbiodiniaceae* through symbiont necrosis and peripheral nuclear chromatin condensation, among other physiological changes (Landsberg *et al*. 2020). This may result from an increase in *rab7* expression among the *Symbiodiniaceae*, which may be signaling for degradation of dead and dysfunctional cells through endocytic phagosomes (Beavers *et al*. 2023). The increased *Symbiodiniaceae* DNA identified in diseased samples in this study may be a byproduct of this necrosis and degradation of the symbiont. This was especially notable in *M*. *meandrites*, which was the coral in this study most susceptible to acute tissue loss and mortality from SCTLD (Precht *et al*. 2016); this accelerated tissue loss may lead to higher levels of dead and dysfunctional symbionts being produced during visual lesion progression in *M*. *meandrites* than in other coral species.

## Conclusions

The novel method employed in this study sheds new light on the microbial dynamics in SCTLD-affected corals and highlights the potential role of *Vibrio* species within the tissue of progressing surface lesions. These findings pave the way for more focused investigations into the role of *Vibrio* and other microbes in SCTLD, which could eventually lead to effective strategies for disease prevention and control. However, the observed associations between certain microbial taxa and SCTLD could be either a cause or a consequence of visual tissue loss. Understanding these dynamics is a crucial aspect of coral disease research that warrants further investigation, along with an understanding of what represents a truly healthy coral microbiome. Apparently healthy corals exposed to SCTLD can experience shifts in their microbiome even in the absence of tissue loss (Huntley *et al*. 2022) and may even be in the early stages of infection with developing subsurface SCTLD lesions (Landsberg *et al*. 2020) emphasizing the need for additional genomic resources for non-model organisms. The lack of comprehensive genomic databases for corals can impede the progress of metagenomic analysis and the identification of potential pathogens. We believe that the continued efforts to sequence and assemble more coral genomes, particularly those affected by SCTLD, would greatly contribute to our understanding of this devastating disease.

## Supplementary Material

1

## Figures and Tables

**Table 1:** Summary of total DNA sequencing reads from each coral species and tissue type. Rows labeled “Filtered” report the unique reads remaining after filtering out reads that overlapped with those found in apparently healthy samples, as described in the text. M=millions of reads.

Coral Species	Tissue Type (# of samples)	Rea Counts (M)
*Diploria labyrinthiformis*	Apparently Healthy (4)	151.125
	Diseased Unaffected (5)	211.401
	Diseased Lesion (5)	198.816
	Filtered Diseased Lesion	0.955
*Dichocoenia stokesii*	Apparently Healthy (5)	179.831
	Diseased Unaffected (5)	202.1 (38
	Diseased Lesion (5)	257.031
	Filtered Diseased Lesion	0.562
*Meandrina meandrites*	Apparently Healthy (3)	126.223
	Diseased Unaffected (5)	206.423
	Diseased Lesion (5)	185.556
	Filtered Diseased Lesion	4.159
*Stephanocoenia intersepta*	Apparently Healthy (5)	197.367
	Diseased Unaffected (5)	190.251
	Diseased Lesion (5)	190.304
	Filtered Diseased Lesion	50.028

## Data Availability

Supplemental materials are available at FigShare. Table S1 contains the average k-mers per read for every genus across all coral species. Table S2 contains the *Symbiodinaceae* protein classifications. Table S3 contains the virulence factors identified in the *Vibrio* contigs. Table S4 contains the alignment rates of reads classified as *Vibrio* to eight draft assemblies of *Vibrio* species previously isolated from SCTLD infected corals. The IDs of the unique diseased reads remaining after filtering for each coral species and the assembled contigs are also available at FigShare. The report files from the KrakenUniq and MMseqs2 analyses have been made available at the following repository: https://github.com/jheinz27/coral_results/tree/main.
