# Supplementary material for: Novel metagenomics analysis suggests a Vibrio species is associated with stony coral tissue loss disease
Source: bioRxiv. 2024 Jan 3:2024.01.02.573916. Preprint. [Version 1] doi: 10.1101/2024.01.02.573916 (PMC10802270; doi:10.1101/2024.01.02.573916)
Supplement: Supplementary file 1 [file NIHPP2024.01.02.573916V1-supplement-1.pdf]

bioRxiv preprint doi: https://doi.org/10.1101/2024.01.02.573916; this version posted January 3, 2024. The copyright holder for this preprint (which was not certified by peer review) is the author/funder, who has granted bioRxiv a license to display the preprint in perpetuity. It is made available under aCC-BY 4.0 International license.

Supplemental Materials

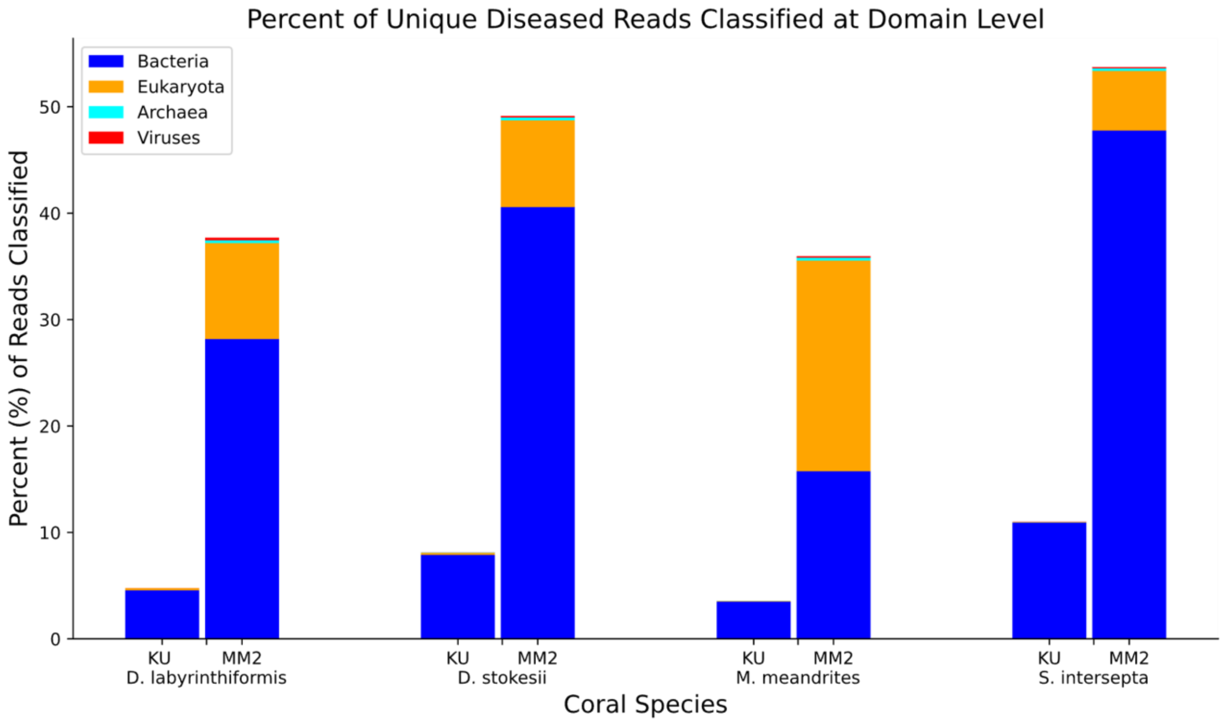

Figure S1: Proportions of unique diseased reads classified by KrakenUniq (KU) and MMseqs2 (MM2) subdivided by the superkingdom they belong to for each coral species.

Table S1- Average k-mers per read for every genus across all coral species.

Table S2- *Symbiodinaceae* proteins that had at least one unique diseased read align to the UniRef50 cluster (paired reads classified separately) for each coral species.

Table S3- Hits to the Virulence Factor Database by assembled contigs that were classified as *Vibrio* by KrakenUniq.

Table S4- Alignment rates of reads classified as *Vibrio* to eight draft assemblies of *Vibrio* species isolated from SCTLD infected corals by Ushijima *et al.* 2020

Supplemental methods

Filtering reads with a healthy coral reference database

A database was for each coral species was created with the command:

bioRxiv preprint doi: https://doi.org/10.1101/2024.01.02.573916; this version posted January 3, 2024. The copyright holder for this preprint (which was not certified by peer review) is the author/funder, who has granted bioRxiv a license to display the preprint in perpetuity. It is made available under aCC-BY 4.0 International license.

```
krakenuniq-build –db pooled_healthy_reads --threads 32 --kmer-len 29
```

Each read k-mer found in the healthy reads was stored under the same taxonomy ID. The diseased lesion reads from the same species were then classified using the created database with the following command:

```
krakenuniq --db database_from_healthy_reads --threads 32 --paired --report
diseased_against_healthy.kuniqreport pooled_1.fasta pooled_2.fasta >
diseased_against_healthy.kuniq
```

Only unclassified reads were kept for downstream analysis.

KrakenUniq read classification

This command generated report files which list the number of reads and unique k-mers for all taxa, including counts at the strain, species, genus, and higher levels.

```
krakenuniq --db /ccb/salz8-4/data/krakendbs/krakendb-2020-08-16-
all_pluseupath/ --threads 16 --paired --report results.kuniqreport
filtered_reads_1.fasta filtered_reads_2.fasta > results.kuniq
```

MMseqs2 read classification

The flowing commands were used to clasify the paired read files individually with the Mmseq2 easy-taxonomy workflow against the UniRef50 database.

```
mmseqs easy-taxonomy filtered_reads_1.fasta uniref50_db
filtered_reads_results_1 tmp --threads 16

mmseqs easy-taxonomy filtered_reads_1.fasta uniref50_db
filtered_reads_results_1 tmp --threads 16
```

The report files were sorted by most abundant families using the command:

```
awk -F'\t' '$4 == "family" {print $0}' results_report | sort -nrk2,2
```
